# School and School‐Related Experiences of Children and Adolescents With Inflammatory Bowel Disease: A Scoping Review

**DOI:** 10.1111/cch.70249

**Published:** 2026-04-09

**Authors:** Jenna Rice, Catherine Wilkinson

**Affiliations:** ^1^ School of Sport and Exercise Sciences Liverpool John Moores University Liverpool UK; ^2^ School of Education Liverpool John Moores University Liverpool UK

**Keywords:** adolescents, children, experiences, IBD, school

## Abstract

**Background:**

This scoping review explores the school and school‐related experiences of children and adolescents with Inflammatory Bowel Disease (IBD), drawing on 10 studies identified from an initial pool of 2676 records published between 2014 and 2022 across eight countries. The review identifies the following four key themes: school attendance/absenteeism; educational outcomes; general school experience; and school functioning and school‐related quality of life outcomes.

**Methods:**

This scoping review included studies that were predominantly quantitative in design, with findings synthesised thematically across study types, alongside a narrative summary of quantitative indicators such as absenteeism, school functioning, and school‐related quality of life.

**Results:**

The findings reveal that, while educational outcomes for children with IBD were generally not significantly different from their peers (although some studies noted lower academic performance in children with IBD), children with IBD often felt that teachers and peers lacked understanding of their condition, which led to increased stress and sometimes bullying. Furthermore, children with IBD had lower quality‐of‐life scores related to school functioning compared with healthy peers. Despite these challenges, participation in school activities varied, with some children missing out on physical education and extracurricular activities.

**Conclusion:**

Overall, the review highlights the need for more research directly exploring children's perspectives on the emotional and embodied impact of IBD on their everyday lived school experiences. Furthermore, it emphasises the importance of improving school accommodations and understanding for children with IBD among peers, teachers and wider school staff. Future studies should consider qualitative approaches, including use of creative methods, to deepen the understanding of the complex lived experiences of schoolchildren with IBD.

## Summary

- Children and adolescents with IBD experience reduced participation in school life, particularly through increased absenteeism and difficulties with school functioning.
- School participation for children with IBD is shaped by interactions between illness‐related factors and modifiable school environments, rather than by disease activity alone.
- Limited understanding of IBD within schools can act as a significant barrier to participation, inclusion and well‐being.
- Existing research focuses largely on attendance and academic outcomes, highlighting a need for child‐centred research that captures lived experiences of participation in school for children with IBD.
- Educational policies and practices should prioritise flexible, inclusive approaches to support meaningful participation for children living with chronic illness.

## 1 Introduction

Inflammatory Bowel Disease (IBD), which includes two major subtypes: Crohn's disease (CD) and ulcerative colitis (UC), is a chronic condition that causes ulceration and inflammation in the gut (Rosen et al. 2015). Approximately 25% of patients with IBD are diagnosed as children and adolescents (Borowitz 2023) with incidence rates continuing to increase (Henderson and Dobson 2024). For children in the United Kingdom, incident rates per 100 000 person‐years have increased from 13.1 (8.4–19.5) in 2000 to 25.4 (19.5–32.40) in 2020 (Pasvol et al. 2020). This upward trend has continued, with an estimated 49 cases per 100 000 in 2023, for children under 16 years old (Henderson and Dobson 2024). IBD can have a significant impact on children's quality of life (Freckmann et al. 2018) and can interfere with psychosocial and physical development (Duricova et al. 2017). In turn, this can have a negative effect on school, potentially compromising educational achievement (Mackner et al. 2012).

Evidence suggests that IBD can have an adverse impact on the lives of children and adolescents. For instance, IBD can negatively impact physical activity and sports participation (Werkstetter et al. 2012), which can have long‐term effects on physical health (i.e., bone growth, strength) and social development (Mählmann et al. 2017). Similarly, IBD can lead to feelings of embarrassment and stress with respect to needing to stay near a bathroom, or activity limitations, in turn affecting self‐esteem through periods of prolonged isolation (Capilla‐Díaz et al. 2019). Evidence also suggests that children with IBD, compared to those without, have poorer psychological well‐being (Mählmann et al. 2017). This is further echoed in research with young adults (15–25 years); for example, Halloran et al. (2021) found that 41% of young adults with IBD reported poor well‐being and over one‐third were at risk of depression, with participants also demonstrating more negative illness perceptions and a lower internal locus of control than peers with other chronic illnesses. Aggregation of these aforementioned stressors can lead to poor mental health. Mental health problems were 28% higher in children and adolescents with IBD, compared to a matched control (Cooney et al. 2024). In the same review, IBD patients were at significant risk of posttraumatic stress disorder, self‐harm and depression, with 31% at risk of developing one or more mental health problem by 25 years old when compared with 25.1% in control groups (Cooney et al. 2024). Moreover, IBD has been shown to negatively impact body image due to delayed puberty (Gasparetto and Guariso 2014), with these feelings already exacerbated during adolescence. Many adolescents place significant value on bodily appearance and physical attractiveness (Barned et al. 2022) and therefore bodily changes associated with medication (i.e., acne, weight loss or gain) can be difficult to process. For some, the presence of stoma bags (waterproof pouches that collect waste from the body) can further impact negative feelings towards oneself, consequently leading some children and adolescents to feel resentment towards their body (Fourie et al. 2018). For these reasons, it is important to understand the school experiences of children and adolescents with IBD to reduce these stressors.

School experiences may be negatively affected by IBD and IBD‐related health issues for children and adolescents (Mackner et al. 2012). Children with IBD attend school less frequently than their healthy peers (Barnes et al. 2020). For instance, Eloi et al. (2019) found that children with IBD were absent for 4.8% of school days during the year, compared to 3.2% for their healthy peers. The main causes of absenteeism for children with IBD are the disease itself, hospital attendance and school difficulties (Eloi et al. 2019; Barnes et al. 2020). Regarding the latter connection, school children may feel unwell during school, have restricted access to toilets, be worried about soiling themselves, be bullied because of their IBD, or have concerns about disclosing their condition (Carter and Carter 2024). Recent qualitative work also highlights that returning to school after absence can create challenges in disease management, school functioning, and peer relationships for adolescents with IBD (Peng et al. 2025), indicating that the transition back into everyday school life from a period of illness‐related absence warrants greater research attention. It should be noted that school attendance represents only one dimension of school participation. Broader participation also includes children's involvement in learning activities, social relationships, and everyday school life. For children with IBD, participation in school is shaped by interactions between health‐related factors (e.g., symptoms, treatment demands) and environmental conditions within schools, such as access to facilities, flexibility of policies and staff, and peer understanding of the condition.

As children and adolescents spend most of their waking time in school, it is important to understand their school experiences. However, no study exploring the embodied (i.e., perception, feelings and actions that shape their bodies and the world around them), emotional (i.e., subjective experience that involves a combination of physiological and behavioural response to a stimulus and can include bodily feelings and judgements), and affective (i.e., an individual's emotional response to a situation) experiences of school life for children with IBD have been conducted. Understanding these experiences in a school context has the potential to, among other aspects, promote inclusion and reduce stigma, support emotional health and address the physical realities of IBD. Given the limited number of qualitative studies exploring the first‐hand school experiences of children with IBD, the existing literature conceptualises ‘school experience’ largely through quantitative indicators such as attendance, school functioning and school‐related quality of life, often reported by parents or via medical records. This review therefore synthesises evidence across quantitative, mixed‐methods and qualitative designs to characterise school participation and school‐related experiences among children and adolescents with IBD. More specifically, this review addressed the following question: What is known about school attendance, school functioning and school‐related experiences among children and adolescents (≤ 18 years) with IBD.

## 2 Methods

The current review was conducted in accordance with the Preferred Reporting Items for Systematic reviews and Meta‐Analyses extension for Scoping Reviews (PRISMA‐ScR; Peters et al. 2020). A scoping review methodology was selected to enable mapping of the breadth and characteristics of evidence examining school attendance, school functioning and school‐related experiences in paediatric IBD across diverse study designs and outcome measures. Given this descriptive and exploratory aim, no formal quality appraisal or risk‐of‐bias assessment was conducted (Peters et al. 2020).

### 2.1 Search Sources and Search Terms

An initial search of the topic area was conducted to gain insight into terms commonly used across different disciplines (e.g., medicine, education, psychology and sociology). Synonyms of terms were also checked using a thesaurus. The search strategy was designed in accordance with the population‐concept‐context (PCC) framework (Peters et al. 2020) and organised into three domains: population (children and adolescents), concept (IBD) and context (school). From this, a comprehensive search strategy was devised, and the search terms were entered as key words: (‘Inflammatory Bowel Disease’ OR ‘Ulcerative colitis’ OR ‘Crohn's disease’) AND (‘Child’ OR ‘Adolescent’ OR ‘Childhood’ OR ‘Children’ OR ‘Youth’ OR ‘Young people’ OR ‘Students’) AND (‘School’ OR ‘Education’ OR ‘School Life’ OR ‘Classroom’ OR ‘School related’).

### 2.2 Database Searches

A scoping review of the literature published over the last 20 years on school and school‐related experiences of children and adolescents with IBD was conducted. In order to find the existing literature between January 2005 and May 2024, a search was initiated in the following three electronic databases: ERIC, Taylor and Francis Journal and SAGE. These databases were selected as we wanted to capture literature with a specific focus on education, schooling and psychosocial outcomes for pupils.

### 2.3 Inclusion and Exclusion Criteria

The inclusion criteria were as follows: (1) articles focusing on children and adolescents (up to 18 years old), (2) articles focusing on school or school‐related experiences of children with IBD, CD or UC, (3) articles in English, (4) peer‐reviewed articles and book chapters and (5) articles published between 2005 and 2024. Exclusion criteria were as follows: (1) articles involving adults, (2) articles where any other conditions/diseases were explored and (3) conference, abstract or dissertations.

### 2.4 Selection Process

Initially, 2676 articles were found using the aforementioned descriptors: ERIC: 7, Taylor and Francis: 1800, SAGE: 850, manual searching of reference lists of included articles and relevant reviews: 19. The title and abstracts of these studies retrieved were screened by J.R., who classified each paper as included or excluded in accordance with the inclusion criteria described above. To enhance transparency and consistency, a second reviewer (C.W.) reviewed all full‐text inclusion decisions. Any disagreements were resolved through discussion. The results of the searches were exported into Endnote and duplicates were removed. Following this, full text copies of 23 studies were then obtained and were read against the full inclusion criteria. Of these 23 studies, 13 were excluded as full text copies were not available (*n* = 2), they did not meet the population age (*n* = 5), study outcome (*n* = 3) and had insufficient focus on the study population (*n* = 4). The remaining 10 studies fully met the inclusion criteria for this review and were subsequently included in the data synthesis. Table 1 presents the 10 final articles selected, with each one described based on the following categories: (1) author and publication year, (2) country of publication, (3) participants, including sample size, (4) study design and methods (quantitative, qualitative or mixed methods) (5) purpose of the study and (6) main findings of the study, including the most significant outcomes and their contributions. Data extraction and thematic synthesis were conducted by J.R., with regular discussion and review with C.W. to support transparency and reflexivity.

**TABLE 1:** Articles included in the scoping review, with a description of each study.

Author (year)	Country	Participants	Study design and methods	Purpose of the study	Main findings
Assa et al. (2015)	France	142 adolescents aged 11–18 years and parent dyads. (43 with CD, 31 with UC, 42 with FAP and 30 healthy)	Quantitative	To assess school absenteeism in IBD compared with healthy controls.	Compared with health age‐matched peers, children with IBD missed significantly more school, due to medical appointments and hospitalisation. Compared with healthy controls, participation in various school and after school activities was significantly reduced for children with IBD.
Barned et al. (2022)	Canada	25 paediatric patients with IBD aged 10–17 years old.	Phenomenological study. Qualitative (interviews)	To explore the challenges of IBD and how Canadian youth respond to them.	Experiences of IBD are multifaceted, with challenges related to diagnosis, managing identify and making sense of change and navigating sociability.
Barnes et al. (2020)	United Kingdom	169 children and adolescents aged 5–17 years old.	Mixed methods	To examine the extent to which IBD affects school/college attendance for children and determine contributing factors for absences.	For children with IBD attendance rate was significantly lower than all children in England. The main issues related to feeling unwell at school, access to toilets, teachers understanding and keeping up with work.
Carreon et al. (2018)	USA	161 children and adolescents aged 11–18 years old.	Secondary analysis of data. Quantitative	To describe school attendance, performance and participation in a sample of adolescents with IBD.	School attendance was poor, with absences related to doctor appointments, hospitalisation and feeling unwell.
Eloi et al. (2019)	France	106 patients with IBD aged 5–18 years. (497 healthy children aged 6–18)	Multicentric study. Quantitative	To analyse prospectively the rate and the causes of school absenteeism covering an entire school year.	Children with IBD missed school more than health controls. The main reason for school absenteeism was digestive disorders, followed by scheduled events (hospitalisation, endoscopy, consolations)
Freckmann et al. (2018)	Germany and Austria	675 children and adolescents with IBD aged 10–15 years and parents.	Cross‐sectional study. Mixed methods	To describe the experience with the situation in school from the perspective of children with IBD and their parents and to examine determinants of school performance.	Low satisfaction was reported by parents in relation to the disease‐related situation at school. Children with IBD had been repeatedly off school for prolonged periods of time and only grade retention was determined by disease related variables.
Mackner et al. (2012)	USA	92 adolescents aged 11–17 years (50 with IBD and 42 healthy)	Cross‐sectional. Quantitative (questionnaires)	To examine school functioning in adolescents with IBD compared with healthy adolescents.	Compared with healthy controls, children with IBD had poorer school functioning, although only significant differences were observed for school absences.
Malmborg et al. (2019)	Sweden	2827 children with IBD < 17 years old.	Cohort study design. Quantitative	To examine the association between childhood‐onset IBD and school results.	Children with IBD had lower final grade point averages but were not a higher risk of qualifying for high school. Children with IBD underperformed although this was characterised by long‐standing disease activity.
Singh et al. (2015)	Canada	337 participants with a diagnosis of IBD prior to the age of 17. (63% CD and 43% UC)	Quantitative	To determine grade 12 academic performance for children with IBD.	No significant differences were observed between children with IBD and without for scores in language arts and mathematics.
Silva et al. (2020)	Brazil	35 patients with IBD aged 3–18 years. (17 with CD, 17 with UC and 62 healthy)	Cross‐sectional, analytical case series study. Quantitative	To evaluate the quality of life of children and adolescents with IBD.	Compared with the health control group, children's with IBD quality of life was significantly lower due to IBD symptoms, fear of using public toilets and greater disease activity.

*Note:* Crohn's disease and ulcerative colitis.

### 2.5 Analysis

Data were synthesised using reflexive thematic analysis following Braun and Clarke's six‐phase approach (Braun and Clarke 2021). The data comprised extracted findings from the results and discussion sections of included studies, including qualitative text and reported quantitative outcomes related to school attendance, school functioning and school‐related quality of life. Quantitative findings (e.g., absenteeism rates, group differences in school functioning scores) were coded based on their conceptual relevance to school participation rather than their statistical properties and were integrated narratively within themes, rather than pooled meta‐analytically. Analytic decisions were documented through memo writing and theme development was discussed, with the second author (C.W.) acting as a critical friend to further provoke reflexivity and understanding of the data within the studies. These themes were then finalised and J.R. and C.W. agreed on them. Extracted data included qualitative findings and reported quantitative outcomes related to school attendance, school functioning and school‐related quality of life. Coding focused on the conceptual meaning of findings in relation to school participation rather than on statistical properties. Codes were iteratively reviewed and organised into themes, which were refined through discussion between authors to support reflexivity and transparency.

## 3 Results

### 3.1 Study Characteristics

The 10 studies located in this review were published between 2014 and 2022 and were conducted in eight countries. United States of America (*n* = 2), France, (*n* = 2), Canada (*n* = 2), United Kingdom (*n* = 1), Sweden (*n* = 1), Germany and Austria (*n* = 1) and Brazil (*n* = 1). Most of the included studies were quantitative (*n* = 7), two were mixed methods and one was qualitative. All studies explicitly stated the percentage of the type of IBD patients had. Participants in the studies ranged in age from 3 to 18 years and the number of participants in the samples ranged from 25 to 2827. The gender composition of the samples was described in all studies, which included both biologically male and female participants. One study explicitly stated the ethnicity of the participants (Carreon et al. 2018). In addition, two studies specified the participants' socioeconomic status (Freckmann et al. 2018; Mackner et al. 2012). Six studies collected data from children and parents concurrently. Two studies (Barned et al. 2022; Singh et al. 2015) collected data from just children and two studies collected data from other participants (e.g., health agencies). The settings in which these studies collected data were hospitals (*n* = 5), IBD participant databases (*n* = 3), and hospital databases (*n* = 2). One of the studies that collected data from hospitals (Silva et al. 2020) also collected data from health participants from schools. The following four main themes were identified: school attendance/absenteeism, educational outcomes, general school experience, and school functioning and school‐related quality of life outcomes. Although most included studies were quantitative, we synthesised findings thematically across study designs and included a narrative summary of quantitative indicators (e.g., absenteeism, school functioning and related outcomes).

## 4 School Attendance/Absenteeism

A dominant theme identified from these studies was school attendance/absenteeism and, more specifically, high levels of school absenteeism among children with IBD compared to their healthy peers (*n* = 6). For example, Mackner et al. (2012) found that in a sample of American children with IBD, they had significantly more full day absences than their healthy peers. Evidence also indicated that 20% of those children missed more than 3 weeks of school, compared with 4% for healthy children and adolescents. Similar findings were observed in a sample of children with IBD in France (Assa et al. 2015). Specifically, children with CD and UC missed 24 and 21 days of school, respectively. This was compared to age‐matched healthy controls who missed 5.1 days of school. In extreme cases of school absenteeism, children with IBD were found to have missed 82.6 days across the school year (Eloi et al. 2019). Notably, some authors found that school absenteeism differed between school years; that is, children with IBD in high school were found to have significantly greater levels of school absenteeism when compared to children with IBD in primary schools (Eloi et al. 2019; Barnes et al. 2020). There was, however, consensus among these authors that there were no significant differences between genders regarding school absenteeism for children with IBD.

In accordance with the above, we identified three subthemes of factors related to school attendance/absenteeism for children with IBD. Most studies (*n* = 5) found IBD‐related symptoms, such as diarrhoea, abdominal pain and rectal bleeding, to be a main determinant of absenteeism (Assa et al. 2015; Eloi et al. 2019; Carreon et al. 2018; Barnes et al. 2020; Barned et al. 2022). Across these studies, authors found these IBD‐related symptoms to account for between 48% and 78% of absenteeism in their respective samples. The second subtheme was planned care. Planned care included hospital and/or doctors' appointments, examinations and consultations and was found to significantly contribute to school absenteeism (Eloi et al. 2019; Assa et al. 2015; Barnes et al. 2020; Carreon et al. 2018). There was evidence across these studies that planned care accounted for between 27% and 78% of absenteeism across the school in the respective studies. School officials emerged as the final subtheme for a factor relating to absenteeism (Barned et al. 2022; Barnes et al. 2020; Freckmann et al. 2018). There was evidence to suggest that school staff were not accommodating in meeting the children's needs and had very limited understanding of IBD and the related symptoms (Barnes et al. 2020; Freckmann et al. 2018). One study further detailed how some schools were not accommodating in allowing children with IBD to access the toilet during school, consequently leading to one child not attending school for the subsequent two years (Barned et al. 2022). There was, however, evidence from one study to suggest that school absenteeism was not currently an issue for some children with IBD, which was partly attributed to the status and management of their IBD (e.g., remission vs. flare) (Barnes et al. 2020).

### 4.1 Educational Outcomes

Another theme identified was the educational outcomes of children with IBD, which was identified by several authors (*n* = 5). Generally, there is evidence across the studies that IBD did not significantly impact educational outcomes (Freckmann et al. 2018; Carreon et al. 2018; Singh et al. 2015; Mackner et al. 2012). For example, in the cross‐sectional study by Freckmann et al. (2018), findings demonstrated that poor educational marks were rare among children with IBD. That is, only 26 out of 675 children with IBD failed mathematics and German in the previous academic term, while 189 out of the 675 children achieved good marks in both subjects. Some further studies demonstrated how parents reported their children had achieved an average or above‐average grade during the last school period (Carreon et al. 2018). Although Singh et al. (2015) found no significant differences in grade 12 educational outcomes among children with or without IBD, findings did suggest that the prevalence of mental health conditions at the time of an IBD diagnosis was a predictor of worse educational outcomes. In contrast, however, and although differences were nonsignificant, evidence suggests that children with IBD do have lower grade point averages compared to healthy children (Mackner et al. 2012). Similar findings were found in other studies. For example, Malmborg et al. (2019) examined school performance in 2827 children with IBD, concluding that these children had lower grade point averages than their respective siblings and reference individuals.

### 4.2 General School Experience

General school experiences for children with IBD emerged as an overarching theme in some studies (*n* = 4). There was evidence in the studies that the quality of life in school was relatively low, although this was not consistent across the studies. Two subthemes were identified, which related to general school experience: (1) satisfaction with the situation at school and (2) participation in school and school‐related activities. It was apparent that across the studies, satisfaction with the situation at school was generally low, with low satisfaction observed for knowledge, attentiveness, and school facilities (Freckmann et al. 2018). From a parental perspective, some studies highlight how they felt that the school was uninterested in their child's condition, emphasising that they felt that their child's disease was irrelevant to them. Parents also felt that educational support during their child's relapse and teachers' knowledge and understanding of IBD was insufficient (Freckmann et al. 2018). In the same study, children's perspectives were also reported. Generally, children felt that teachers had a limited understanding of their condition and, as a result, would not ask them about it. Children also felt like other pupils did not fully understand their condition, particularly during periods where they appeared weak and fatigued because of medication taken to manage their IBD (Freckmann et al. 2018). Some studies further detailed how the initial stress before school and during school intensified their distress and made them feel angry about their condition. Finally, the lack of understanding by teachers in school about IBD and their reluctance to allow children with IBD to use the toilet during and between scheduled lessons resulted, for some children, in them having accidents in school. For one child, this led to them being bullied in school by peers (Barned et al. 2022).

Regarding the second subtheme, findings related to participation in school and school‐related activities are presented. There was evidence that children with IBD struggled to keep up with their homework. For example, Carreon et al. (2018) found that 40% of parents reported difficulties with their child with IBD keeping up with schoolwork, while only 11% had recent difficulty with reading or doing their homework. One study noted that children and adolescents with IBD had to sacrifice participation in meaningful activities, such as participation in school sports, due to their condition (Barned et al. 2022). Similar findings were observed by Assa et al. (2015), who found children with IBD were less likely than their healthy peers to participate in school activities such as gym classes and after‐school sport activities. In the same study, there was also evidence that there was no significant difference between children with IBD and healthy peers in relation to social activities in school (i.e., school trips) and out‐of‐school social activities (Assa et al. 2015). In contrast, Freckmann et al. (2018) found that some parents reported that their child struggled going on school trips and when they found the courage to attend, they were taken back early due to panic attacks and diarrhoea, a key IBD symptom. There was also evidence to suggest that schools were reluctant, and in some instances refused, for children with IBD to participate in exchange programmes because of their condition (Freckmann et al. 2018).

## 5 School Functioning and School‐Related Quality of Life Outcomes

A final theme, although less prevalent across the included studies, highlighted the impact of IBD on school functioning and school‐related quality of life outcomes (Mackner et al. 2012; Silva et al. 2020). Across the included studies, psychosocial outcomes were operationalised primarily as *school functioning* (e.g., attention/concentration, keeping up with work) and *school‐related quality of life* (quality of life school functioning domains). Few studies directly measured mental health outcomes within school contexts; where mental health conditions were reported, they were typically examined as correlates or predictors of educational outcomes rather than as school‐based endpoints. Compared with healthy controls, children with IBD had poorer school functioning (Mackner et al. 2012). This included paying attention in class, forgetting things, keeping up with schoolwork, and missing school because of illness or going to visit the doctor and/or hospital. However, the authors noted that there was only a significant difference between healthy controls and children with IBD for school absences. In another study, Silva et al. (2020) found that school functioning for children with IBD was significantly lower compared to healthy peers (Silva et al. 2020). Furthermore, when children experienced symptom onset below the age of 2, the differences become even more significant. In relation to psychosocial scores, children with IBD also scored significantly lower compared to healthy peers (Silva et al. 2020). Similarly to school functioning, these differences become more pronounced when symptoms onset before the age of 2. Some studies found similar results, suggesting that the quality of school life for children with IBD was significantly lower than those of healthy peers.

## 6 Discussion

This scoping review explored the school and school‐related experiences of children and adolescents with IBD. Overall, the findings suggest that school experiences for children with IBD are shaped by a complex interaction of illness‐related, psychosocial and environmental factors. These include the nature and current state of the disease (e.g., remission vs. flare), the presence and severity of symptoms such as abdominal pain, diarrhoea, fatigue and rectal bleeding, and the extent to which these symptoms are accommodated within the school environment. Together, these factors influence school attendance, engagement and broader participation in school life. School attendance emerged as a key area of difficulty, particularly for older children and adolescents attending secondary school (Barnes et al. 2020; Schwenk et al. 2014). Increased absenteeism was commonly attributed to disease flares, medical appointments and hospitalisation. However, while several studies quantified school absence, they provided little insight into children's experiences of returning to school following periods of illness‐related absence, including how missed learning, social disruption and anxiety are managed. This represents an important gap in the literature, as returning to school may pose distinct challenges that are not captured by attendance data alone.

Recent qualitative work begins to address this gap. A study by Peng et al. (2025) explored adolescents' experiences of returning to school after IBD‐related absence and identified ongoing challenges associated with symptom management within the school environment, impaired school functioning, and difficulties maintaining friendships. These findings are particularly salient during adolescence, a developmental period in which peer relationships and social belonging are central to well‐being (Yang et al. 2025). For young people with IBD, physical symptoms and emotional concerns such as embarrassment, fear of stigma, or anxiety around disclosure, may act as barriers to forming and sustaining friendships, compounding the impact of illness on school participation. These experiences can be situated within the broader literature on schooling and chronic illness, which highlights how health‐related absences, symptom unpredictability and environmental barriers shape children's participation and experiences at school (Jay et al. 2025). For children with IBD, these challenges may be further exacerbated by the often‐invisible nature of symptoms and limited understanding of the condition among school staff and peers (Rice and Wilkinson 2025). Viewed through the lens of the International Classification of Functioning, Disability and Health (ICF), school attendance and engagement can be conceptualised as forms of participation that arise from interactions between health conditions and contextual factors, rather than as direct consequences of illness alone (World Health Organisation 2007). Difficulties sustaining peer relationships may therefore have implications not only for social well‐being but also for school engagement, identity development and long‐term educational participation among adolescents living with IBD.

The review also highlights the importance of the school environment itself in shaping the experiences of children with IBD. Several studies reported that school staff often lack understanding of IBD, leading to inadequate accommodations and limited support (Schwenk et al. 2014; Barnes et al. 2020). In some cases, children were denied access to essential facilities such as toilets during lessons or between classes, which exacerbated symptoms and contributed to emotional distress. These findings illustrate how everyday school policies and practices can act as significant barriers to participation for children managing a gastrointestinal condition (Wilkinson and Budworth 2025). Although some studies reported no significant impact of IBD on academic performance, the psychosocial aspects of school life emerged as particularly important. Children with IBD frequently reported lower satisfaction with their school experience, citing difficulties participating in school activities, lack of understanding from peers and teachers, and insufficient support as contributing factors (Mackner et al. 2012; Schwenk et al. 2014). These findings suggest that academic attainment alone does not fully capture the impact of IBD on children's educational experiences, as children may continue to achieve academically while experiencing emotional or social difficulties at school.

It is also important to consider the role of contextual and structural differences across countries represented in this review. The included studies span eight countries with differing educational systems, healthcare models and approaches to supporting children with chronic illness in school. Variations in access to healthcare, flexibility of school attendance policies, availability of learning support services and legal requirements for school accommodations may all shape children's experiences of attending school with IBD (Heijink et al. 2017; Kaarsen 2014). As this scoping review aimed to map the breadth of evidence rather than compare national systems, these contextual influences could not be examined systematically. However, future research would benefit from explicitly exploring how educational and healthcare contexts interact to influence school participation and support for children with IBD.

Comparisons with other chronic illnesses further contextualise these findings. Research consistently shows that children with chronic illnesses are more likely to experience school absence and disruption than their healthy peers (Jay et al. 2025). However, evidence suggests that children with gastrointestinal conditions, including IBD, may report particularly high levels of unmet school‐related needs and dissatisfaction with school support compared to children with other chronic conditions, such as diabetes or asthma (Bechara et al. 2018; Langton et al. 2020). This may reflect the unpredictable and socially sensitive nature of IBD symptoms, which can be difficult to accommodate within standard school routines (Wilkinson and Budworth 2025). At the same time, similarities across chronic conditions are evident, including challenges related to keeping up with schoolwork, maintaining peer relationships and managing stigma (Lum et al. 2017; Runions et al. 2020). Together, these findings highlight the need for both condition‐specific understanding and broader inclusive approaches to supporting children with chronic illnesses in school settings.

An important contribution of this scoping review is its examination of how school‐related issues in paediatric IBD have been studied. Of the 10 included studies, the majority were quantitative, focusing primarily on attendance, educational outcomes and school‐related quality of life. While these studies provide valuable insight into the scale and distribution of school‐related difficulties, they are limited in their ability to capture the complexity of children's everyday lived experiences at school. The relative lack of child‐centred qualitative research means that children's own perspectives on managing symptoms, navigating social relationships and coping with school demands remain underrepresented. Where qualitative data are included, they are often secondary to quantitative measures or rely on proxy reporting from parents, which may obscure aspects of school life that are most salient to children themselves. This highlights the need for further qualitative and mixed‐methods research that prioritises children's voices and explores school experiences in greater depth.

## 7 Strengths and Limitations

This scoping review provides an overview of how school attendance, school functioning and school‐related experiences in paediatric IBD have been examined across diverse study designs and outcomes. By synthesising evidence from quantitative and qualitative studies, the review identifies key patterns and gaps in the literature. As the purpose of the review was descriptive, formal quality appraisal was not conducted. Findings should therefore be interpreted as a mapping of existing research rather than as an evaluation of the strength or quality of individual studies.

## 8 Conclusion and Future Directions

This scoping review highlights clear patterns and gaps in how school attendance, school functioning and school‐related experiences in paediatric IBD have been examined. The existing literature places strong emphasis on quantitative indicators such as absenteeism and educational outcomes. While valuable, this focus provides limited insight into the emotional, social and embodied experiences of school life for children and adolescents with IBD. There remains a notable lack of research that directly captures children's everyday lived experiences of attending school with this condition. Where qualitative research has been conducted, it has largely relied on traditional interview‐based methods, with little use of creative or participatory approaches that may be more accessible and meaningful for children.

Future research should prioritise in‐depth qualitative exploration of children's school experiences and consider the use of creative and participatory research methods, such as drawing (Noonan et al. 2016), diaries or participatory approaches (Rice and Wilkinson 2025), alongside care‐full methods (Budworth 2023; Wilkinson 2025), which have been shown to support children in expressing embodied and emotional aspects of chronic illness experiences. Such methods may generate insights that are directly relevant to educational practice, including the design of flexible attendance policies, bathroom access arrangements and peer and teacher education initiatives to support inclusion for children with IBD. In addition, none of the studies included in this review explicitly examined online, hybrid or distance education as a mode of schooling for children with IBD. Given the increasing availability of digital learning and its potential relevance during periods of illness or treatment, this represents an important area for future research. Addressing these gaps will be essential for developing support that promotes meaningful school participation and well‐being for children and adolescents living with IBD.

## Author Contributions

**Jenna Rice:** methodology (lead), writing – original draft (lead), writing – review and editing (equal). **Catherine Wilkinson:** conceptualization (lead), writing – review and editing (equal).

## Funding

Funding was received from British Educational Research Association (BERA SGA2025—to C.W.).

## Ethics Statement

Ethical approval was not required for this study as it is a scoping review of previously published literature and did not involve the collection of primary data from human participants or animals.

## Data Availability

The authors have nothing to report.

## References

[cch70249-bib-0001] Assa, A. , A. Ish‐Tov , F. Rinawi , and R. Shamir . 2015. “School Attendance in Children With Functional Abdominal Pain and Inflammatory Bowel Diseases.” Journal of Pediatric Gastroenterology and Nutrition 61, no. 5: 553–557. 25950089 10.1097/MPG.0000000000000850

[cch70249-bib-0002] Barned, C. , A. Fabricius , A. Stintzi , D. R. Mack , and K. C. O'Doherty . 2022. “The Rest of my Childhood Was Lost: Canadian Children and Adolescents' Experiences Navigating Inflammatory Bowel Disease.” Qualitative Health Research 32, no. 1: 95–107. 34818940 10.1177/10497323211046577 PMC11951367

[cch70249-bib-0003] Barnes, C. , J. J. Ashton , F. Borca , M. Cullen , D. M. Walker , and R. M. Beattie . 2020. “Children and Young People With Inflammatory Bowel Disease Attend Less School Than Their Healthy Peers.” Archives of Disease in Childhood 105, no. 7: 671–676. 31937567 10.1136/archdischild-2019-317765

[cch70249-bib-0004] Bechara, G. M. , F. Castelo Branco , A. L. Rodrigues , et al. 2018. “KiDS and Diabetes in Schools Project: Experience With an International Educational Intervention Among Parents and School Professionals.” Pediatric Diabetes 19, no. 4: 756–760. 29504189 10.1111/pedi.12647

[cch70249-bib-0005] Borowitz, S. M. 2023. “The Epidemiology of Inflammatory Bowel Disease: Clues to Pathogenesis?” Frontiers in Pediatrics 10: 1103713. 36733765 10.3389/fped.2022.1103713 PMC9886670

[cch70249-bib-0006] Braun, V. , and V. Clarke . 2021. “One Size Fits All? What Counts as Quality Practice in (Reflexive) Thematic Analysis?” Qualitative Research in Psychology 18, no. 3: 328–352.

[cch70249-bib-0007] Budworth, P. 2023. “Care, Comfort and Capacity: The Importance of Being Flexible in Research With Disabled and Chronically Ill People.” SSM 4: 100352.

[cch70249-bib-0008] Capilla‐Díaz, C. , C. Bonill‐de Las Nieves , S. M. Hernández‐Zambrano , et al. 2019. “Living With an Intestinal Stoma: A Qualitative Systematic Review.” Qualitative Health Research 29, no. 9: 1255–1265. 30678525 10.1177/1049732318820933

[cch70249-bib-0009] Carreon, S. A. , L. T. Bugno , A. A. Wojtowicz , and R. N. Greenley . 2018. “School Functioning in Adolescents With Inflammatory Bowel Diseases: An Examination of Disease and Demographic Correlates.” Inflammatory Bowel Diseases 24, no. 8: 1624–1631. 29718311 10.1093/ibd/izy026

[cch70249-bib-0010] Carter, J. , and S. Carter . 2024. “Family Reflections: Research Gives Back Childhood: A Family's Experience With Very Early Onset Inflammatory Bowel Disease.” Pediatric Research 97, no. 1: 442–443. 39251882 10.1038/s41390-024-03506-8

[cch70249-bib-0011] Cooney, R. , D. Tang , K. Barrett , and R. K. Russell . 2024. “Children and Young Adults With Inflammatory Bowel Disease Have an Increased Incidence and Risk of Developing Mental Health Conditions: A UK Population‐Based Cohort Study.” Inflammatory Bowel Diseases 30, no. 8: 1264–1273. 37603846 10.1093/ibd/izad169 PMC11291622

[cch70249-bib-0012] Duricova, D. , M. Fumery , V. Annese , P. L. Lakatos , L. Peyrin‐Biroulet , and C. Gower‐Rousseau . 2017. “The Natural History of Crohn's Disease in Children: A Review of Population‐Based Studies.” European Journal of Gastroenterology & Hepatology 29, no. 2: 125–134. 27748673 10.1097/MEG.0000000000000761

[cch70249-bib-0013] Eloi, C. , G. Foulon , L. Bridoux‐Henno , et al. 2019. “Inflammatory Bowel Diseases and School Absenteeism.” Journal of Pediatric Gastroenterology and Nutrition 68, no. 4: 541–546. 30418416 10.1097/MPG.0000000000002207

[cch70249-bib-0014] Fourie, S. , D. Jackson , and H. Aveyard . 2018. “Living With Inflammatory Bowel Disease: A Review of Qualitative Research Studies.” International Journal of Nursing Studies 87: 149–156. 30125834 10.1016/j.ijnurstu.2018.07.017

[cch70249-bib-0015] Freckmann, M. , A. Seipp , M. W. Laass , et al. 2018. “School‐Related Experience and Performance With Inflammatory Bowel Disease: Results From a Cross‐Sectional Survey in 675 Children and Their Parents.” BMJ Open Gastroenterology 5, no. 1: e000236. 10.1136/bmjgast-2018-000236 PMC6254744 30538821

[cch70249-bib-0016] Gasparetto, M. , and G. Guariso . 2014. “Crohn's Disease and Growth Deficiency in Children and Adolescents.” World Journal of Gastroenterology 20, no. 37: 13219–13233. 25309059 10.3748/wjg.v20.i37.13219 PMC4188880

[cch70249-bib-0017] Halloran, J. , B. McDermott , T. Ewais , et al. 2021. “Psychosocial Burden of Inflammatory Bowel Disease in Adolescents and Young Adults.” Internal Medicine Journal 51, no. 12: 2027–2033. 32840949 10.1111/imj.15034

[cch70249-bib-0018] Heijink, R. , P. Reitmeir , and R. Leidl . 2017. “International Comparison of Experience‐Based Health State Values at the Population Level.” Health and Quality of Life Outcomes 15, no. 1: 138. 28683747 10.1186/s12955-017-0694-9 PMC5501450

[cch70249-bib-0019] Henderson, P. , L. Dobson , and PINPOINT Collaborators . 2024. “OC3 Pinpoint: The Prospective Epidemiology of Paediatric‐Onset Inflammatory Bowel Disease in the UK–A Prospective, National, Cohort Study”.

[cch70249-bib-0020] Jay, M. A. , A. Zylbersztejn , L. Herlitz , J. Deighton , R. Gilbert , and R. Blackburn . 2025. “Chronic Health Conditions and School Absence, Exclusions and Non‐Enrolment: A Cohort Study Using the Education and Child Health Insights From Linked Data Database.” Journal of Public Health 47: fdaf064. 10.1093/pubmed/fdaf064 PMC12395948 40455597

[cch70249-bib-0021] Kaarsen, N. 2014. “Cross‐Country Differences in the Quality of Schooling.” Journal of Development Economics 107: 215–224.

[cch70249-bib-0022] Langton, C. R. , J. P. Hollenbach , T. Simoneau , and M. M. Cloutier . 2020. “Asthma Management in School: Parents' and School Personnel Perspectives.” Journal of Asthma 57, no. 3: 295–305. 10.1080/02770903.2019.1568455 30676162

[cch70249-bib-0023] Lum, A. , C. E. Wakefield , B. Donnan , M. A. Burns , J. E. Fardell , and G. M. Marshall . 2017. “Understanding the School Experiences of Children and Adolescents With Serious Chronic Illness: A Systematic Meta‐Review.” Child: Care, Health and Development 43, no. 5: 645–662. 28543609 10.1111/cch.12475

[cch70249-bib-0024] Mackner, L. M. , R. M. Bickmeier , and W. V. Crandall . 2012. “Academic Achievement, Attendance and School‐Related Quality of Life in Pediatric Inflammatory Bowel Disease.” Journal of Developmental & Behavioral Pediatrics 33, no. 2: 106–111. 22267107 10.1097/DBP.0b013e318240cf68

[cch70249-bib-0025] Mählmann, L. , M. Gerber , R. I. Furlano , et al. 2017. “Psychological Wellbeing and Physical Activity in Children and Adolescents With Inflammatory Bowel Disease Compared to Healthy Controls.” BMC Gastroenterology 17: 1–10. 29233119 10.1186/s12876-017-0721-7 PMC5727963

[cch70249-bib-0026] Malmborg, P. , N. Mouratidou , M. C. Sachs , et al. 2019. “Effects of Childhood‐Onset Inflammatory Bowel Disease on School Performance: A Nationwide Population‐Based Cohort Study Using Swedish Health and Educational Registers.” Inflammatory Bowel Diseases 25, no. 10: 1663–1673. 30916332 10.1093/ibd/izz040

[cch70249-bib-0027] Noonan, R. J. , L. M. Boddy , S. J. Fairclough , and Z. R. Knowles . 2016. “Write, Draw, Show and Tell: A Child‐Centred Dual Methodology to Explore Perceptions of Out‐of‐School Physical Activity.” BMC Public Health 16, no. 1: 326. 27080384 10.1186/s12889-016-3005-1 PMC4832535

[cch70249-bib-0028] Pasvol, T. J. , L. Horsfall , S. Bloom , et al. 2020. “Incidence and Prevalence of Inflammatory Bowel Disease in UK Primary Care: A Population‐Based Cohort Study.” BMJ Open 10, no. 7: e036584. 10.1136/bmjopen-2019-036584 PMC7371214 32690524

[cch70249-bib-0039] Peng, J. , Y. Yuan , J. Zhang , Y. Ding , and X. He . 2025. “Global, Regional and National Burden of Inflammatory Bowel Disease in Females From 1990 to 2021: An Analysis of the Global Burden of Disease Study 2021.” Frontiers in Global Women's Health 6: 1580451. 10.3389/fgwh.2025.1580451 PMC12158981 40510919

[cch70249-bib-0042] Peters, M. D. , C. Marnie , A. C. Tricco , et al. 2020. “Updated Methodological Guidance for the Conduct of Scoping Reviews.” JBI Evidence Synthesis 18, no. 10: 2119–2126. 33038124 10.11124/JBIES-20-00167

[cch70249-bib-0029] Rice, J. , and C. Wilkinson . 2025. “The Everyday School Experiences of Children and Young People With Inflammatory Bowel Disease.” Education 3‐13: 1–14.

[cch70249-bib-0030] Rosen, M. J. , A. Dhawan , and S. A. Saeed . 2015. “Inflammatory Bowel Disease in Children and Adolescents.” JAMA Pediatrics 169, no. 11: 1053–1060. 26414706 10.1001/jamapediatrics.2015.1982 PMC4702263

[cch70249-bib-0031] Runions, K. C. , R. Vithiatharan , K. Hancock , et al. 2020. “Chronic Health Conditions, Mental Health and the School: A Narrative Review.” Health Education Journal 79, no. 4: 471–483.

[cch70249-bib-0032] Schwenk, H. T. , J. R. Lightdale , J. H. Arnold , D. A. Goldmann , and E. R. Weitzman . 2014. “Coping With College and Inflammatory Bowel Disease: Implications for Clinical Guidance and Support.” Inflammatory Bowel Diseases 20, no. 9: 1618–1627. 25105948 10.1097/MIB.0000000000000124

[cch70249-bib-0033] Silva, L. C. , R. B. M. Seixas , and E. de Carvalho . 2020. “Quality of Life in Children and Adolescents With Inflammatory Bowel Disease: Impact and Predictive Factors.” Pediatric Gastroenterology, hepatology & nutrition 23, no. 3: 286. 10.5223/pghn.2020.23.3.286 PMC7231741 32483550

[cch70249-bib-0034] Singh, H. , Z. Nugent , M. Brownell , L. E. Targownik , L. L. Roos , and C. N. Bernstein . 2015. “Academic Performance Among Children With Inflammatory Bowel Disease: A Population‐Based Study.” Journal of Pediatrics 166, no. 5: 1128–1133. 25598305 10.1016/j.jpeds.2014.12.010

[cch70249-bib-0036] Werkstetter, K. J. , J. Ullrich , S. B. Schatz , C. Prell , B. Koletzko , and S. Koletzko . 2012. “Lean Body Mass, Physical Activity and Quality of Life in Paediatric Patients With Inflammatory Bowel Disease and in Healthy Controls.” Journal of Crohn's and Colitis 6, no. 6: 665–673. 10.1016/j.crohns.2011.11.017 22398103

[cch70249-bib-0041] Wilkinson, C. 2025. “Towards Painless and Productive Research Relationships: Reflections on Study Design by a Researcher With Chronic Pain for Participants With Chronic Pain.” Frontiers in Pain Research 5: 1450667. 39974654 10.3389/fpain.2024.1450667 PMC11835849

[cch70249-bib-0040] Wilkinson, C. , and P. Budworth . 2025. “Pooh‐Poohing School Toilet Policies: (More Than) Leaky Bodies as Resistance for School Children With Inflammatory Bowel Disease (IBD).” Children's Geographies 23, no. 3: 413–420.

[cch70249-bib-0037] World Health Organization . 2007. International Classification of Functioning, Disability and Health: Children & Youth Version: ICF‐CY. World Health Organization.

[cch70249-bib-0038] Yang, Y. , Y. Jin , Y. Wang , and C. Wu . 2025. “Place Attachment Enhances Adolescents' Subjective Well‐Being via Basic Psychological Needs Satisfaction in the Context of Peer Rejection.” Journal of Environmental Psychology 108: 102837.

